# Lipoma of the Uterine Corpus: Exceptional Eventuality Combined with an Ovarian Thecoma

**DOI:** 10.1155/2009/340603

**Published:** 2009-05-26

**Authors:** R. Vilallonga, A. García, J. Castellví, J. M. Fort, M. Armengol, S. Ramón y Cajal

**Affiliations:** ^1^Surgery Department, Endocrinology and Metabolic Unit, Hospital Vall d'Hebron, 08035 Barcelona, Spain; ^2^Pathology Department, Endocrinology and Metabolic Unit, Hospital Vall d'Hebron, 08035 Barcelona, Spain

## Abstract

Uterine lipomas are very uncommon with symptoms that are similar to leiomyomas. Their diagnosis is always histological although some radiological methods may suggest their existence prior to surgery. They are sometimes associated with endometrial pathology, but there are no previous reported cases related to ovarian thecoma. Their prognosis is excellent. Clinical, radiological, morphologic, and immunohistochemical findings are shown which correspond to uterine lipoma associated with endometrial polyps and ovarian thecoma.

## 1. Introduction

Uterine lipomas are extraordinarily rare entities [1–3]. The presence of fat in the uterine corpus is not exceptional and, in fact, it is known that some leiomyomas have an adipose tissue component, in variable proportions, associated to smooth muscular fibre. These cases are known as lipoleiomyomas [4, 5] and certain authors consider them to be hamartomatous lesions [6]. On the other hand, lipomas are those tumours that are exclusively comprised of mature adipose tissue. The clinical manifestations do not usually differ greatly to those caused by leiomyomas except that they affect women that are somewhat older and normally postmenopausal [1]. Most lipomas are located in the body of the uterine corpus and the size can range from a few millimetres up to masses of more than several dozen centimetres in diameter. Diagnosis is accomplished after a meticulous analysis of the surgical piece, although some radiological techniques may indicate their existence prior to surgery [7]. Their prognosis is excellent, except in cases associated to malignant intracavitary pathology [8]. We present a case associated to endometrial polyps and ovarian fibroma.

## 2. Clinical Case

**Figure 1:**
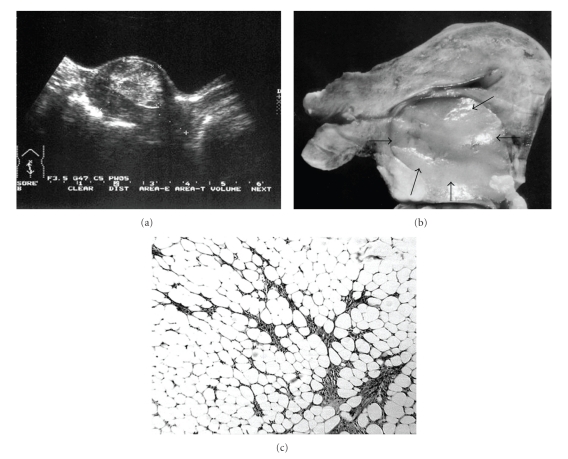
(a) Suprapubic ultrasound study with full bladder (+). Longitudinal cut of the uterus with a well-defined nodular lesion (arrows), intramural and hyperechogenic on the anterior uterine face. (b) Surgical resection piece: anteroposterior surface cut. Presence of a well-defined intramural nodular lesion in the anterior face with a surface that is homogeneous, bright, and yellowish in colour (arrows). An endometrial polyps can also be seen in the uterine fundus. (c) Microscopic image of the uterine lipoma. Solid masses of mature adipocytes occasionally delimited by fibroconnective tracts with capillary vessels. Hematoxilin-eosin, 200X.

A 48-year-old woman with no clinical history of interest, and gynaecological history of menarche at 14-years-of-age, regular menstrual cycles with a duration of 5 days every 28 days. Parity of 2-0-0-2. She is currently climacteric with amenorrhoea for 6 months and has not received any hormone replacement therapy. She presented with clinical symptoms of pelvic pain in the right iliac fossa region of 3 months' evolution. Initially, the patient was evaluated for acute appendicitis, tubal pregnancy, and other inflammatory processes. That pain was occasional, dull, not related to movements, and sometimes referred as a puncture. A suprapubic abdominal ultrasound scan showed an enlarged uterine corpus that measured 140 × 65 × 43 mm and, in anteversion, a well-defined hyperechogenic 52 × 32 mm nodular intramural formation was highlighted on the anterior face (Figure 1(a)), as well as an endometrial polyps in the uterine fundus with a maximum diameter of 20 mm. Uterine adnexa were not seen because of the uterine size. Using Doppler, the uterine wall nodule appeared avascular. With the diagnosis of hypertrophic and myomatous uterine corpus, the patient was submitted to surgery for total hysterectomy and bilateral adnexectomy. Patient was previously evaluated and was considered that the suitable treatment should be one only surgical procedure. For this reason, endometrial polypectomy was not performed previously. The postoperative evolution was without complications and with subsequent remission of the pelvic pain. Patient had endovenous treatment for the pain during three days and after oral treatment for 5 more days. At 1 year follow-up, patient only refers some skin paresthesias and no abdominal pain. 

The pathological study revealed a hypertrophic uterine corpus of 140 grams and 14 × 7 × 5 cm. Dissection revealed an intramural tumorous development of 52 × 32 mm, which bulged into the endometrial cavity, with a smooth cut surface and somewhat elastic consistency and yellowish coloration (Figure 1(b)). Microscopically, it was almost entirely comprised of mature adipocytes occasionally defined by fibrous tracts containing capillary vessels (Figure 1(c)). Peripherally, the lesion was surrounded by a fibrous capsula. By using immunohistochemical techniques, positivity was observed for markers such as vimentine, and focally for actin in the smooth muscle of the fibroconnective tracts around the capillary vessels. On the contrary, other markers such as CD10, CD99, CD34, and HMB45 were negative. The remaining findings were centred on a 20 × 3 mm endometrial polyps implanted in the uterine fundus with a histology characteristic of glands with irregular morphology, some cystic, on a dense stroma rich in thick-walled vessels. The left ovary was 80 × 55 mm, of hard consistency with a yellowish, trabecular surface. Microscopically, it consisted of fibroblasts, partly vacuolar, on a hyaline stroma, edematous and occasionally collagenized, characteristic of ovarian thecomas.

## 3. Discussion

Lipomas of the uterine corpus are extraordinarily rare entities and, since they were first described by Lopstein in 1816 [9], somewhat less than 20 cases have been published in their pure form. With the exception of some works like those of Krenning [1], Willén [5], or Sieinski [10] on a series of lipomatous lesions (leiomyomas with a fatty component), most of these tumours are reflected in literature as isolated events. Their clinical manifestation is identical to that caused by leiomyomas. More than half the cases first present with uterine bleeding, and a somewhat smaller proportion, as in the case of our patient, with abdominal pain when these tumours reach larger dimensions. Pain is rare as a unique symptom and the pain referred by our patient is difficult to explain with these findings. Lipomas of the uterine corpus usually appear in women with a higher mean average age than is common for leiomyomas, mainly after the menopause [1, 11], with most being postmenopausal; our patient was 48-years-of-age at the time of diagnosis. All cases were diagnosed after histological analysis of the surgical piece, although some works have also appeared in which certain radiological techniques could indicate their existence before surgery [7]. Seraph and cols. [12] performed ultrasonographic analyses in 11 lipomatous lesions of the uterine corpus which they observed as avascular masses with well-defined hyperechogenic margins. In our case, the ultrasound study also reflected these findings, including emphasised tumour avascularization by using Doppler colour. US identifies a hyperechoic mass sometimes encased in a hypoechoic ring. The uterin myolipoma can be a mixed (fat and solid tissue) uterine mass in CT scan. However, diagnosis may be difficult if the mass is exophytic or pedunculated. On T( 2)-weighted MR images, fat content within the tumour can be confirmed because of evident chemical shift artefact [13].

Certain association have been seen between uterine lipomas and endometrial polyps [1], and more rarely with carcinoma [8, 14]. However, we do have to consider that the lipoma, polyp, and thecoma were just coincidental. It is very difficult to demonstrate any kind of relation between these three entities in our same patient.

The role that these lesions may play in the genesis of endometrial neoplasias is not well known, although some authors point to a hypothetical androgen to oestrogen conversion in the fat of the lipoma itself [1]. With respect to the associated ovarian pathology, some cases of hemangioma have been described [11] but, until now, never thecomas as in the case of our patient. Similarly, we do not know to what extent the hormonal factors of these ovarian neoplasias could be involved with lipoma physiopathology. 

Differential histological diagnosis does not present great difficulties for the recognition of a lesion, which is principally or exclusively comprised of mature adipocytes. Nevertheless, they could present serious problems in those rare cases in which lipomas are located submucosally [7], and where the only material available is a small specimen obtained by hysteroscopy. In these circumstances, the most frequent entities to discard would be lipoleiomyomas, clear cell leiomyomas and liposarcomas as an integral part of mixed Müllerian tumours. In most cases immunohistochemical techniques and the absence of atypias would resolve these reasonable doubts. 

The genesis of these lesions in the uterine wall continues to be an enigma. It is not really known how a fatty tumour can develop where fatty tissue did not previously exist under normal conditions. Many theories have been proposed, such as migration of adipose cells through the uterine arteries [15]; mesenchymal cellular remains of a pluripotential nature with the capacity to differ toward adipocytes [16], or adipose metaplasia from the smooth muscle fibre component of the uterine corpus [10]. This latter seems to enjoy greater acceptance and there are in fact some works published that stand by the use of immunohistochemistry, demonstrating positivity for smooth muscle actin in part of the adipose population [11]. In our case, immunohistochemical techniques were also performed in paraffin, emphasising smooth muscle actin positivity in perivascular mesenchymal cells and negativity for CD10, CD99, CD34, and HMB45, thus partly reinforcing this theory. 

In conclusion it can be said that lipomas of the uterine corpus are extraordinarily rare entities with some clinical manifestations similar to leiomyomas, they have intuitive radiological characteristics, demonstrative histology and excellent prognosis.
